# Neurophysiological changes following intensive Naikan therapy in a patient with recurrent depressive episodes later diagnosed with bipolar II disorder: A case report

**DOI:** 10.1002/pcn5.70444

**Published:** 2026-11-15

**Authors:** Atsushi Furuichi, Yuko Higuchi, Naohito Kaneko, Tsutomu Takahashi

**Affiliations:** ^1^ Tanino Clinic, Medical Corporation Wakeikai Toyama City Toyama Japan; ^2^ Department of Neuropsychiatry University of Toyama Graduate School of Medicine and Pharmaceutical Sciences Toyama City Toyama Japan; ^3^ Research Center for Idling Brain Science University of Toyama Toyama City Toyama Japan

**Keywords:** bipolar II disorder, Naikan therapy, P300, resting‐state EEG, sLORETA

## Abstract

**Background:**

Intensive Naikan therapy is a structured Japanese psychotherapy involving systematic reflection on interpersonal relationships; however, its neurophysiological correlates remain unclear. We herein report changes in resting‐state electroencephalography (EEG), the P300 event‐related potential, and standardized low‐resolution brain electromagnetic tomography (sLORETA)–estimated source activity after intensive Naikan therapy.

**Case Presentation:**

A 29‐year‐old man with recurrent depressive episodes and anxiety about family relationships and future employment underwent 7 days of inpatient intensive Naikan therapy. He was later diagnosed with bipolar II disorder after a hypomanic episode during the follow‐up. Assessments were performed before and after the intervention. Anxiety decreased on State–Trait Anxiety Inventory state and trait scores (42 to 20 and 30 to 20, respectively), and depressive symptoms remained minimal on the Hamilton Depression Rating Scale and Beck Depression Inventory (4 to 3 and 0 to 0, respectively). Psychological measures suggested reduced self‐ and other‐blame and greater acceptance of family members. Resting‐state EEG showed a negligible change in peak alpha‐band global field power, whereas peak beta‐band global field power decreased from 5.13 × 10^3^ to 2.84 × 10^3^ µV^2^/Hz. During the auditory oddball task, P300 amplitude at Pz increased from 5.5 to 7.3 µV. sLORETA images were evaluated descriptively, without statistical comparisons or uncertainty estimations.

**Conclusion:**

Intensive Naikan therapy was temporally associated with reduced resting‐state beta activity and an increased P300 amplitude. These observations from a single uncontrolled case with multiple assessments are hypothesis‐generating and do not establish treatment efficacy, causality, or a treatment mechanism.

## BACKGROUND

Naikan therapy, a Japanese psychotherapy involving systematic reflection on interpersonal relationships, has been used to alleviate psychological distress and as an adjunctive intervention for psychiatric symptoms.^1^ Clinical studies have suggested its benefits for depressive and anxiety‐related symptoms.^2^, ^3^ However, the cited evidence includes an uncontrolled pre–post study on 28 patients with anxiety disorders^2^ and a non‐randomized comparison of daily Naikan practice in 47 patients with depression, all of whom had received intensive Naikan therapy.^3^ These designs limit attribution of the reported benefits specifically to intensive Naikan therapy.

Only a few studies have examined physiological changes during Naikan therapy. Shiga serially recorded frontal electroencephalographic (EEG) activity, electromyography (EMG), and palm skin temperature in three participants during 7 days of intensive Naikan therapy.^4^ The findings obtained showed more sustained mid‐alpha activity, decreased EMG, and increased skin temperature, possibly reflecting relaxation and adaptation to the Naikan setting. Nevertheless, standardized pre–post EEG studies using quantitative measures, event‐related potentials, and source localization are limited.

We previously reported the clinical course and psychodynamic formulation of the present case,^5^ but not the neurophysiological findings. We herein present exploratory changes in resting‐state EEG, P300, and standardized low‐resolution brain electromagnetic tomography (sLORETA)–estimated source activity before and after intensive Naikan therapy in a patient with recurrent depressive episodes who was later diagnosed with bipolar II disorder.

## CASE PRESENTATION

### Patient information and clinical course

At the time of intensive Naikan therapy, the patient was a 29‐year‐old unmarried man with recurrent depressive episodes, anxiety about the future, and longstanding family‐related interpersonal difficulties, particularly involving his father. In his early 20s, he developed insomnia, loss of appetite, nausea, depressed mood, and anxiety. He initially received treatment for a somatoform disorder and was subsequently treated for depression.

Despite multiple antidepressant trials and repeated hospitalizations for recurrent symptoms and self‐harm, symptoms persisted, and he was referred to our hospital for consideration of electroconvulsive therapy (ECT). At admission, depressive symptoms were mild, with a Hamilton Depression Rating Scale (HAM‐D) score of 14 and a Beck Depression Inventory (BDI) score of 16. Depressive symptoms improved during inpatient rest and supportive listening, and ECT was not performed.

However, low self‐confidence and concerns about family relationships and future employment persisted. Consistent with these interpersonal difficulties, a personality assessment using the Minnesota Multiphasic Personality Inventory (MMPI)^6^ suggested low stress tolerance, difficulty expressing emotions appropriately, and passive‐aggressive tendencies (Supplement 1). Since the psychosocial assessment indicated persistent family‐related difficulties, intensive Naikan therapy was initiated on hospital Day 40 after psychoeducation.

### Intensive Naikan therapy

Intensive Naikan therapy was conducted for seven consecutive days (hospital Days 40–46) in a private room on the ward as an inpatient adaptation of the original Naikan method.^7^ The patient systematically reflected on significant others using three standard themes: (1) what he had received from the person, (2) what he had given to the person, and (3) the trouble and difficulties he had caused them. The procedure and concomitant medication are detailed in Supplement 2.

During the first several days, he was able to recall theme‐congruent episodes, although his descriptions were brief and non‐specific. His recollections from the mid‐phase onward became more concrete, particularly regarding his mother. He also increasingly described events from others' perspectives, including reframing his father's scolding as guidance intended to teach right from wrong. By the final day, he expressed a more agentic attitude toward his life. He acknowledged that he had not sufficiently confronted his illness and stated that he wanted to engage more actively in treatment and efforts toward social reintegration.

### Clinical and psychological assessments

Intellectual functioning was assessed at admission using the Wechsler Adult Intelligence Scale‐Revised.^8^

After obtaining consent, clinical and psychological assessments were conducted before intensive Naikan therapy (Pre‐INT; hospital Day 36) and after the completion of intensive Naikan therapy (Post‐INT; hospital Day 50). Depressive and anxiety symptoms were assessed using HAM‐D,^9^ BDI,^10^ and State–Trait Anxiety Inventory.^11^ Interpersonal‐cognitive and coping‐related changes were evaluated using the Picture‐Frustration Study,^12^ Sentence Completion Test (SCT; Seiken adult version),^13^ and Coping Inventory for Stressful Situations.^14^ The SCT was reviewed descriptively by the treating psychiatrist and a clinical psychologist without formal scoring. Quantitative results are summarized in Table 1.

**Table 1:** Clinical assessments and psychometric measures.

	At admission	Pre‐INT (Day 36)	Post‐INT (Day 50)
HAM‐D	14	4	3
BDI	16	0	0
STAI			
S‐anxiety	‐	42	20
T‐anxiety	‐	30	20
WAIS‐R			
VIQ	76		
PIQ	91		
FSIQ	80		
P‐F study (%)			
Extraggression	‐	44	39
Intraggression	‐	31	23
Imaggression^a^	‐	25	38
CISS			
Problem‐oriented coping	‐	43	75
Emotion‐oriented coping	‐	53	29
Avoidance‐oriented coping	‐	35	59

Abbreviations: ‐, not assessed; BDI, Beck Depression Inventory; CISS, Coping Inventory for Stressful Situations; FSIQ, Full Scale Intelligence Quotient; HAM‐D, Hamilton Depression Rating Scale; INT, intensive Naikan therapy; P‐F study, Picture‐Frustration Study; PIQ, Performance Intelligence Quotient; Pre‐INT, before intensive Naikan therapy; Post‐INT, after intensive Naikan therapy; S‐anxiety, state anxiety; STAI, State–Trait Anxiety Inventory; T‐anxiety, trait anxiety; VIQ, Verbal Intelligence Quotient; WAIS‐R, Wechsler Adult Intelligence Scale‐Revised.

^a^ Imaggression refers to impunitive or non‐blame responses.

### Neurophysiological assessment

Resting‐state EEG, the P300 event‐related potential, and sLORETA source estimation were performed on hospital Days 35 and 49 (around 2:00 p.m., respectively), before and after intensive Naikan therapy, based on a previously published protocol.^15^ EEG was recorded using a 32‐channel DC amplifier (EEG‐2100 version 2.22J, Nihon Kohden Corp., Tokyo, Japan) with electrodes placed according to the international 10–20 system (Fp1, Fp2, F3, F4, C3, C4, P3, P4, O1, O2, F7, F8, T7, T8, P7, P8, Fz, Cz, and Pz). Recordings were conducted in a wave‐shielded and sound‐attenuated room. The sampling rate was 500 Hz, with a bandwidth filter of 0.16–120 Hz and a 60‐Hz notch filter. The reference electrodes were averaged ear electrodes, and electrode impedance was kept below 10 kΩ.

In resting‐state EEG, the patient lay on a bed with his eyes closed in a silent, darkened room for 5 min. EEG data for the resting‐state EEG analysis were segmented into consecutive 2‐s epochs. Epochs with voltage amplitudes exceeding ±200 μV were rejected. After rejection, 89 and 79 epochs were retained for Pre‐INT and Post‐INT recordings, respectively. These epochs were then imported into sLORETA software. Using the “EEG to cross spectrum” function, cross‐spectral matrices were computed over a frequency range of 1–20 Hz. Global field power (GFP) was calculated from 19 scalp electrodes, and the resulting GFP spectra were obtained. The alpha‐ and beta‐frequency ranges were defined according to the 2017 International Federation of Clinical Neurophysiology (IFCN) terminology: alpha activity was defined as 8–13 Hz and beta activity as frequencies >13 Hz.^16^

The P300 component was elicited using an auditory oddball paradigm. Standard tones of 1000 Hz were presented with a probability of 80% (*n* = 200), and target tones of 2000 Hz with a probability of 20% (*n* = 50). Auditory stimuli were presented binaurally through headphones. Each tone had a duration of 100 ms, with a rise/fall time of 10 ms and a variable interstimulus interval of 1.5–2.5 s. The patient was instructed to remain awake, keep his eyes open, fixate on a red circle displayed on a monitor, attend to the auditory stimuli, and press a button as quickly and accurately as possible in response to target tones. P300 data were analyzed using Vital Tracer and EPLYZER II (Kissei Comtec Co., Ltd., Nagano, Japan). EEG epochs of 700 ms were extracted, with a baseline period from −100 to 0 ms before stimulus onset. Epochs with voltage amplitudes exceeding ±50 μV were manually rejected. After artifact rejection, 41 and 39 artifact‐free target epochs were retained for the Pre‐INT and Post‐INT assessments, respectively, and were averaged offline to obtain the P300 waveform. P300 amplitude and latency were measured at Pz, where P300 amplitude is typically maximal. Within the 250–400‐ms time window after target stimulus onset, the positive maximum peak was identified.

In the source estimation, P300 waveforms were analyzed using the “EEG/ERPs to sLORETA” function in sLORETA software. Current source density (CSD) was estimated from the 19 scalp electrodes included in the analysis within the 250–400‐ms post‐target‐stimulus time window, corresponding to the period in which the P300 component was observed. The estimated CSD distribution was then visualized as sLORETA images.

### Pre–post assessment findings

Clinical, psychological, and neurophysiological findings from Pre‐INT to Post‐INT are summarized in Table 1, Figure 1, and Supplement 3. Anxiety decreased, while depressive symptoms remained minimal. Psychological measures suggested reduced blame‐related responses, decreased emotion‐oriented coping, increased problem‐oriented coping, and more accepting SCT narratives toward family members. Resting‐state EEG showed a negligible change in peak alpha‐band GFP, whereas peak beta‐band GFP decreased from 5.13 × 10^3^ to 2.84 × 10^3^ µV^2^/Hz (Supplement 3). P300 amplitude increased at the Pz electrode from 5.5 to 7.3 µV (Figure 1a). sLORETA source estimates are presented descriptively in Figure 1b.

**Figure 1:**
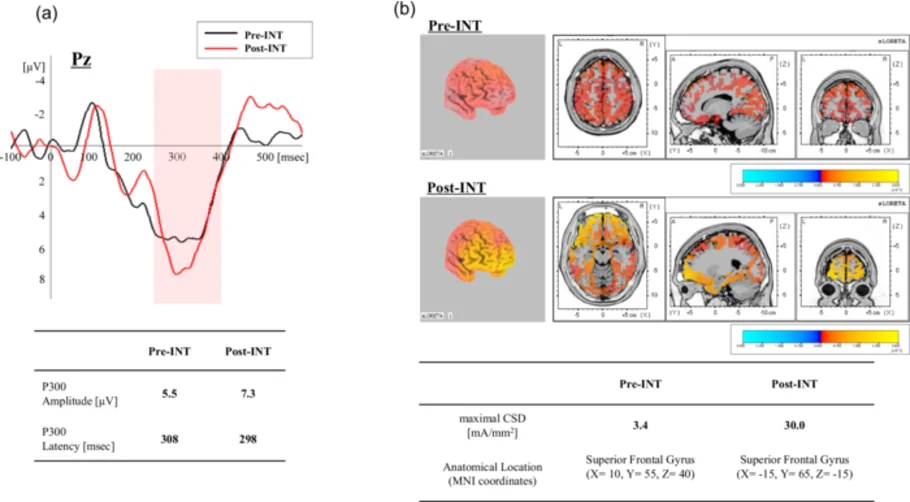
P300 and standardized low‐resolution brain electromagnetic tomography (sLORETA) findings at before intensive Naikan therapy (Pre‐INT) (hospital Day 35) and after intensive Naikan therapy (Post‐INT) (hospital Day 49). (a) P300 waveforms recorded at Pz. Black and red lines represent Pre‐INT and Post‐INT recordings, respectively. The shaded area indicates the P300 analysis time window (250–400 ms). (b) sLORETA source images of P300 within the defined time window. Upper panels show Pre‐INT recordings, and lower panels show Post‐INT recordings. The same color scale was applied to Pre‐INT and Post‐INT sLORETA images. Abbreviations: CSD, current source density; MNI, Montreal Neurological Institute.

### Follow‐up and diagnostic revision

The patient was discharged on hospital Day 53 after improvements in depressive and anxiety symptoms and more considerate interactions with family members. Approximately 1 year later, he experienced a hypomanic episode. Antidepressants were discontinued, mood stabilizers were optimized, and the diagnosis was revised to bipolar II disorder according to the Diagnostic and Statistical Manual of Mental Disorders, Fourth Edition, Text Revision, which was in use at that time.^17^ In the present study, the longitudinal diagnostic course was retrospectively reviewed and confirmed to meet the criteria for bipolar II disorder in the Diagnostic and Statistical Manual of Mental Disorders, Fifth Edition.^18^ He subsequently returned to work.

## DISCUSSION

In the present case, beta‐band GFP decreased, and P300 amplitude increased after intensive Naikan therapy, whereas the change in alpha‐band GFP was negligible. sLORETA images were evaluated descriptively. Anxiety and blame‐related responses decreased, and SCT narratives suggested greater acceptance toward family members, consistent with previous findings of reduced externalized blame^19^ and changes in empathy‐related indices after Naikan therapy.^20^ To the best of our knowledge, this is the first study to examine P300 and sLORETA‐estimated source activity before and after intensive Naikan therapy. However, concomitant medication, inpatient care, sleep and vigilance, repeated testing, and the evolving diagnostic context preclude attributing these observations specifically to Naikan therapy.

The relative stability of alpha‐band GFP does not exclude differences in wakefulness or drowsiness during resting‐state EEG recordings between Pre‐INT and Post‐INT. The reduction in beta‐band GFP occurred alongside lower anxiety scores, whereas depressive symptoms were already minimal at Pre‐INT. For context, increased beta‐band power and decreased alpha1‐band power have been reported in generalized anxiety disorder.^21^ In a different clinical context, Higuchi et al. reported a relationship between frontal β1 activity and somatic concern in drug‐free subjects with at‐risk mental states.^22^ These findings raise the hypothesis that beta‐band GFP may be related to anxiety; however, this relationship was not established in the present case. A cross‐disorder review found marked overlap and within‐disorder variability in resting‐state EEG band‐power findings, as well as methodological heterogeneity.^23^ Accordingly, the beta‐band difference observed here is not sufficient to identify a specific clinical process. Although Shiga reported different physiological findings during intensive Naikan therapy in healthy participants,^4^ differences in the study design, measures, and clinical context preclude direct comparisons.

sLORETA images were evaluated descriptively for the P300 time window before and after intensive Naikan therapy. However, because this was a single‐case report and no statistical comparisons or uncertainty estimations were performed for sLORETA images, the results obtained cannot be interpreted as evidence of increased frontal cortical activity. With this caution in mind, the post‐intervention increase in P300 amplitude at Pz is a descriptive observation of unknown functional significance. P300 abnormalities have been reported in schizophrenia, bipolar disorder, and major depressive disorder and, thus, are not unique to a single diagnosis.^24^ Treatment‐associated P300 changes have also been reported; however, methodological heterogeneity limits comparisons across studies.^25^ P300 amplitude is affected by attention, arousal, and stimulus/task parameters.^26^ Therefore, an amplitude increase alone does not identify a treatment‐specific effect or a particular cognitive improvement.

The later diagnosis of bipolar II disorder changes the diagnostic context in which these findings need to be interpreted. Possible contributions from a bipolar‐spectrum pathophysiology or illness‐related state variation cannot be distinguished from any potential effects of Naikan therapy. Therefore, these results cannot be generalized to patients with major depressive disorder. This case highlights the importance of a longitudinal diagnostic assessment when interpreting neurophysiological observations in patients with recurrent depressive episodes.

There are several limitations that need to be addressed. This was a single uncontrolled case. Changes across multiple measures in the same patient do not constitute the independent replication of a treatment effect. Furthermore, with only one pre‐intervention and one post‐intervention neurophysiological recording session, the stability and reproducibility of the observed differences remain unknown. Inpatient rest, supportive care, expectancy, and the passage of time cannot be separated from any potential intervention‐specific effects. Although EEG recordings were performed under conditions that were kept as consistent as possible, including the recording time and analytic procedures, the pre‐ and post‐intervention assessments were conducted on different days. Therefore, we cannot completely exclude the possibility that day‐to‐day variability or other uncontrolled factors (including sleep, vigilance, fatigue, and repeated exposure to the oddball task) may have contributed to the observed neurophysiological changes. Additionally, the patient received multiple psychotropic medications, including several benzodiazepine‐type agents (Supplement 2). Benzodiazepine administration has been associated with increased EEG beta power and a reduced P300 amplitude.^27^ Although the regimen remained unchanged between Pre‐INT and Post‐INT, unchanged doses do not establish equivalent drug effects at both recordings. Therefore, medication‐related effects cannot be excluded, and their contribution to the observed pre–post differences remains unclear. Since it was not possible to retrieve behavioral response data during the P300 task, including the accuracy of button‐press responses to target stimuli, we were unable to investigate whether pre–post differences in task performance contributed to the observed P300 amplitude difference. Although the retained target‐epoch counts were similar (41 and 39), possible contributions from trial‐count differences or residual noise cannot be excluded. Detailed neuropsychological testing was also not available. Moreover, sLORETA provides an indirect low‐resolution estimate of cortical source activity.

## CONCLUSION

This case illustrates neurophysiological changes before and after intensive Naikan therapy in a patient with recurrent depressive episodes later diagnosed with bipolar II disorder. Reduced beta‐band GFP and an increased P300 amplitude occurred alongside improvements in anxiety and interpersonal‐cognitive measures. These uncontrolled observations across multiple measures are descriptive and hypothesis‐generating, do not establish treatment efficacy, causality, or a treatment mechanism, and require replication.

## AUTHOR CONTRIBUTIONS

Atsushi Furuichi treated the patient and drafted the manuscript. Yuko Higuchi conducted EEG recordings and analyzed EEG data, drafted the neurophysiological methods section, and critically reviewed and commented on the manuscript. Naohito Kaneko contributed to the analysis of EEG data. Tsutomu Takahashi critically reviewed and commented on the manuscript. All authors read and approved the final manuscript.

## CONFLICT OF INTEREST STATEMENT

The authors declare no conflicts of interest.

## ETHICS APPROVAL STATEMENT

Ethical review and approval were not required for this case report according to institutional policy.

## PATIENT CONSENT STATEMENT

Written informed consent was obtained from the patient for the publication of this case report, including the clinical course, interventions, examinations, and accompanying clinical, psychological, neurophysiological, and medication data.

## CLINICAL TRIAL REGISTRATION

N/A.

## Supporting information

Supplement_Revision 1.

## Data Availability

Data that support the results of this case report are available from the corresponding author upon reasonable request. Behavioral response data from the auditory oddball task are not available. The data are not publicly available due to privacy and ethical restrictions.
