# Supplementary material for: Neurophysiological changes following intensive Naikan therapy in a patient with recurrent depressive episodes later diagnosed with bipolar II disorder: A case report
Source: PCN Rep. 2026 Nov 15;5(4):e70444. doi: 10.1002/pcn5.70444 (PMC13770293; doi:10.1002/pcn5.70444)
Supplement: Supplementary file 1 [file PCN5-5-e70444-s001.docx]

**Supplementary Material**

**Supplement 1. Minnesota Multiphasic Personality Inventory profile at admission Supplement 2. Procedure for intensive Naikan therapy in the present case**

**Supplement 3. Resting-state EEG global field power (GFP) at Pre-INT and Post-INT**

**Supplement 1. Minnesota Multiphasic Personality Inventory profile at admission**

The vertical axis indicates standardized T-scores, and the horizontal axis shows the three validity scales and ten clinical scales of the MMPI.

**Abbreviations:** MMPI, Minnesota Multiphasic Personality Inventory; L, Lie; F, Infrequency; K, Correction; Hs, Hypochondriasis; D, Depression; Hy, Hysteria; Pd, Psychopathic Deviate; Mf, Masculinity–Femininity; Pa, Paranoia; Pt, Psychasthenia; Sc, Schizophrenia; Ma, Hypomania; Si, Social Introversion.

**Supplement 2. Procedure of intensive Naikan therapy in the present case**

**Intervention structure and Naikan themes**

Intensive Naikan therapy was conducted as a seven-day inpatient intervention (hospital days 40–46) in a private room on the ward. During the intervention, the patient was asked to reflect systematically on his relationships with significant others. Reflection was organized chronologically from early childhood to the present, and each relationship was reviewed in 3- to 5-year blocks. In each period, reflection was structured around three standard Naikan themes: (1) what he had received from the person, (2) what he had given to the person, and (3) the trouble and difficulties he had caused that person.

**Sequence of reflection and therapeutic aim**

In the present case, reflection began with his mother, followed by his father and sibling. After completing reflections on family members, he also reflected on other important individuals, including close friends and workplace supervisors. The procedure was intended to promote the concrete recollection of interpersonal experiences and re-examination of self–other relationships from multiple perspectives.

**Interview process and therapeutic stance**

Brief interviews with the attending psychiatrist were held approximately every 1–2 hours. During each interview, the patient summarized the content of his reflection for approximately 5–10 minutes. The clinician listened in an accepting and supportive manner and did not provide interpretive comments or direct the patient toward particular conclusions. When the patient experienced difficulty engaging in Naikan, the clinician provided brief process-oriented encouragement. The clinician acknowledged that reflection may feel painful, explained that continuing the process may be therapeutically meaningful, and encouraged him to gradually increase the time he was able to concentrate on Naikan.

**Treatment setting and concomitant medication**

Except for meals and basic self-care, the patient was encouraged to focus on Naikan practice and to avoid unnecessary social interaction and leisure activities. The following psychotropic medications, prescribed by the referring psychiatrist, were continued at the same daily doses throughout intensive Naikan therapy: lithium carbonate 400 mg/day, carbamazepine 400 mg/day, clonazepam 2 mg/day, fluvoxamine maleate 100 mg/day, mianserin hydrochloride 30 mg/day, levomepromazine 25 mg/day, promethazine 25 mg/day, flunitrazepam 2 mg/day, and brotizolam 0.25 mg/day. No psychotropic medication was initiated, discontinued, or dose-adjusted between Pre-INT and Post-INT.

**Supplement 3. Resting-state EEG global field power (GFP) at Pre-INT and Post-INT**

Recordings were obtained on hospital days 35 (Pre-INT) and 49 (Post-INT). **Abbreviations**: EEG, electroencephalography; GFP, global field power; Pre-INT, before intensive Naikan therapy; Post-INT, after intensive Naikan therapy.
